# Analysis of the Mediating Role of Self-Efficacy and Self-Esteem on the Effect of Workload on Burnout’s Influence on Nurses’ Plans to Work Longer

**DOI:** 10.3389/fpsyg.2018.02605

**Published:** 2018-12-18

**Authors:** María del Mar Molero, María del Carmen Pérez-Fuentes, José Jesús Gázquez

**Affiliations:** ^1^University of Almería, Almería, Spain; ^2^Universidad Autónoma de Chile, Santiago, Chile

**Keywords:** self-efficacy, self-esteem, workload, burnout – professional, psychology, mediating model

## Abstract

At the present time, we know that there is a positive relationship between self-efficacy and self-esteem in which positive beliefs about one’s own efficacy increase one’s sense of self-worth as stressful situations of a heavy workload are coped with successfully, and this, in turn, affects the nurses’ plans to work longer. Analyze the mediating role of self-efficacy and self-esteem in the effect of workload, measured as the number of users attended to during a workday, on burnout in nursing professionals. A sample of 1307 nurses aged 22 to 60 years who were administered the *Brief Burnout Questionnaire*, the *General Self-Efficacy Scale*, and the *Rosenberg Self-Esteem Scale*, and workload, measured as the number of users attended to during the workday. The results show that professionals with high levels of self-efficacy also scored higher on global self-esteem. Burnout correlated negatively with both variables (self-efficacy and self-esteem). Three clusters were found with the variables (self-efficacy, self-esteem, and workload) showing significant differences in burnout scores among clusters. Self-efficacy and self-esteem function as buffers of the negative effects of workload on burnout. Organizations should design interventions for promoting the personal resources of their workers through training activities and organizational resources (e.g., redesigning job positions) to promote satisfaction and wellbeing of employees, making their stay at work greater.

## Introduction

The World Health Organization [WHO] considers burnout, which has also been analyzed in education (Martos et al., 2018; Vizoso-Gómez and Arias-Gundín, 2018), an occupational illness of special relevance (World Health Organization [WHO], 2016). This syndrome is characterized by gradual physical and mental exhaustion of individuals, feelings of detachment and of negative attitudes toward their job, and perception of diminished professional efficacy (Maslach et al., 2001).

Even though workers in different occupational sectors may suffer from this syndrome, there is a stronger risk for healthcare professionals, because permanent contact with the suffering and illnesses of others makes their work setting particularly emotionally and psychologically stressful (Adriaenssens et al., 2015; Hunsaker et al., 2015; Banerjee et al., 2016). We can’t forget that this syndrome is also present in other sectors, such as in Alzheimer’s Patient Family Caregivers with no Specialized Training (Pérez-Fuentes et al., 2017). Therefore, the importance of its study stems from the negative consequences it has for the health of workers and the organization. For example, it has been demonstrated that burnout is related to a diversity of physical illnesses (musculoskeletal, cardiovascular, gastrointestinal, respiratory infections, etc.) (Jaworek et al., 2010; Kim et al., 2011) and psychological problems (mood, depression and anxiety disorders, etc.) (Bianchi et al., 2015; Maslach and Leiter, 2016), negatively affecting job performance and leading to absenteeism (Schaufeli et al., 2009; Bakker and Demerouti, 2014).

One of the theoretical models of reference in research on wellbeing and job stress is the *Job Demands-Resources Model* (JD-R), developed by Demerouti et al. (2001), to provide an understanding of burnout and other psychological processes that take place in organizations. Among other matters, this model identifies the job demands that are the best predictors of burnout though a process of deterioration of an employee’s health, which can trigger psychosocial distress, absenteeism and lack of worker commitment to the organization (LePine et al., 2005; Bakker et al., 2014; Bakker and Demeoruti, 2017). The job demands which have received the most attention in the literature are related to tasks and functions in job positions, especially workload (Garcia-Izquierdo and Rios-Risquez, 2012; Cooper et al., 2016; Kandelman et al., 2017; Purohit and Vasava, 2017). Workload may be understood from its quantitative perspective, referring to the perception of an excess volume of work with regard to the time available for it, and its qualitative dimension, which alludes to the quality and complexity of work to be done (French et al., 1982). Kalisch and Lee (2014), for example, found that workload, referring to the number of patients attended to in a workday, was related to dissatisfaction of nursing professionals. Meanwhile, Van Bogaert et al. (2017) found that factors related to daily work routines influenced the perception of workload of nursing employees, especially, the large number of patients and severity of illnesses. Nevertheless, these authors suggested that the negative perception of workload is not exclusively determined by the volume of work, but also by the feelings of frustration generated by not being able to attend adequately to the needs of patients or offer them quality service.

In addition, in a recent extension of the original JD-R model, workers’ personal resources were included to complete the structure of the Work Resources and Demands Model (Xanthopoulou et al., 2007). From this perspective, positive self-evaluations or beliefs workers have about their control over their setting can buffer the negative impact of work demands and at the same time, relate positively to engagement and job performance (Bakker and Demeoruti, 2017). Among these beliefs, self-efficacy and worth, which have been widely studied in Organizational Psychology because of their involvement in wellbeing and occupational health, are emphasized (Ventura et al., 2015; Alharbi et al., 2016; Barbaranelli et al., 2018).

Self-efficacy is a “belief” that individuals have about their capacity to control their surroundings and influences the way they behave, think and feel about future events (Bandura, 1977, 1997). In this sense, workers’ beliefs about their self-efficacy are essential to how they perceive the context in which they work, especially when they have to cope with very demanding and potentially stressful job demands (Grau et al., 2012; Ventura et al., 2015). In such cases, the employees with positive beliefs about their self-efficacy respond adaptively to job stressors, predicting positive states of spiraling gains (e.g., engagement) (Ventura et al., 2006; Lorente et al., 2014; Pérez-Fuentes et al., 2018). On the contrary, those workers who consider themselves ineffective, will attribute failures to a deficit in their competence, increasing their feeling of inefficacy (Van Wingerden et al., 2017).

Self-esteem is the global positive or negative evaluation a person has of their self-worth (Rosenberg, 1965). High levels of self-esteem have been related to wellbeing, satisfaction (Orth et al., 2012; Extremera and Rey, 2018) and effective management of stress and coping with conflictive situations (Bajaj et al., 2016; Yildirim et al., 2017).

There is also considerable attention to the study of self-esteem due to its significant repercussions in the school (Pérez-Fuentes and Gázquez, 2010; González-Cabanach et al., 2017) and at work (Bakker et al., 2014; Lin et al., 2018), and more specifically, with regard to the burnout syndrome (Molero et al., 2018a,b).

Finally, we start from the following hypothesis, that some studies have shown that there is a positive relationship between self-efficacy and self-esteem (Maggiori et al., 2016), in which positive beliefs about one’s efficacy increase the feeling of self-worth as stressful situations are coped with successfully (Caprara et al., 2010, 2013).

Our objective was to analyze the mediating role of self-efficacy and self-esteem on the effect of work load, measured as the number of users attended to in the workday, on burnout in nursing professionals.

## Materials and Methods

### Participants

The original sample consisted of 1601 nurses in Andalusia (Spain) randomly selected from different health centers, of whom those actively employed at the time data were acquired were selected. Cases of random answers or incomplete questionnaires were discarded. Thus the final study sample was composed of a total of 1307 participants. The mean age was 32.03 years (*SD* = 6.53) in a range of 22 to 60. Of the total sample, 84.5% (*n* = 1104) were women and 15.5% (*n* = 203) men, with a mean age of 32.03 (*SD* = 6.50) and 32.01 (*SD* = 6.71), respectively. As for their employment situation, 67.1% (*n* = 877) were working at temporary jobs and 32.9% (*n* = 430) had permanent contracts.

### Instruments

An *ad hoc* questionnaire was prepared for sociodemographic data (age, sex), as well as for information on workload, measured as the number of users attended to in a workday.

#### Cuestionario Breve de Burnout [Brief Burnout Questionnaire]

This consists of 21 items on a five-point Likert-type response scale, which evaluates background, elements and consequences of the syndrome (Moreno et al., 1997). Its purpose is an overall evaluation of burnout as well as its background and consequences, in the three blocks the questionnaire is organized in. In the study subject of this paper, the block made up of the three syndrome factors in the Maslach and Jackson model (1981) was used. Instrument reliability for the study sample, specifically, for global burnout, was α = 0.78.

#### General Self-Efficacy Scale

This scale consists of 10 items with a four-point Likert-type format that evaluate a person’s perception of their own competence for managing different stressful situations effectively (Baessler and Schwarcer, 1996). Sanjuán et al. (2000), analyzed the reliability of the scale, finding a Cronbach’s alpha of 0.87. In our case, the calculation of internal consistency of the scale found an alpha of 0.92.

#### Rosenberg Self-Esteem Scale

This was developed for evaluating self-esteem in adolescents (Rosenberg, 1965). It is made up of 10 items whose contents concentrate on feelings of respect and acceptance of oneself. The response is rated on a four-point Likert scale (from 1 = Strongly agree to 4 = Strongly disagree). Other studies have demonstrated its adequate psychometric characteristics in both a general population (Atienza et al., 2000) and in more specific populations (Vázquez et al., 2013). In our case internal consistency was α = 0.86.

### Procedure

Before collecting data, participants were guaranteed compliance with confidentiality and ethical information standards in data processing. The study was approved by the Bioethics Committee of the University of Almería (Spain). Questionnaires were implemented on a Web platform which enabled participants to fill them out online. For control of random or incongruent answers, a series of control questions were inserted and any such cases were discarded from the study sample.

### Data Analysis

First, to explore the relationships between variables, correlation analyses were done for the continuous quantitative variables. A two-stage cluster analysis was also carried out to group participants by self-esteem as a categorical variable (low, medium and high), and other continuous quantitative variables, such as general self-efficacy and the number of users attended to per workday. Once the clusters or groups had been identified, an ANOVA was done to determine the existence of significant differences between groups with respect to burnout as the dependent variable. To determine which groups were significantly different from each other, the *post hoc* Scheffé comparison test was applied. The SPSS statistical software version 23.0 for Windows was used for these analyses.

Finally, a multiple mediation analysis was done with two mediator variables forming a causal chain to compare the mediating effect of the perceived Self-efficacy and Self-esteem variables. The Preacher and Hayes (2008) SPSS macro for mediation effects was used to compute the mediation model. Bootstrapping was applied with coefficients estimated from 5000 bootstraps to test the indirect effect.

## Results

### Burnout in Nursing and Its Relationship With Perceived Self-Efficacy, Self-Esteem, and Workload

Table 1 presents the descriptive statistics of each of the variables of the study and bivariate correlations. The correlation coefficients found reveal that professionals with high levels of self-efficacy also showed higher scores on global self-esteem (*r* = 0.53; *p* < 0.001). Moreover, burnout correlated negatively with both variables (self-efficacy: *r* = -0.19; *p* < 0.001 and self-esteem: *r* = -0.28; *p* < 0.001).

**Table 1:** Burnout, perceived self-efficacy, self-esteem, and workload.

	1	2	3	4	*M*	*SD*	*Skewness*	*Kurtosis*
1. Burnout	–				55.42	7.49	0.632	1.473
2. Perceived self-efficacy	-0.19***	–			31.40	4.57	-0.128	0.842
3. Self-esteem	-0.28***	0.53***	–		26.10	3.75	-0.370	-0.039
4. Workload^(a)^	0.13***	0.07**	0.03	–	20.82	17.28	1.425	2.193

*Correlations and descriptive statistics (N = 1307). ^(a)^Number of users attended to in a workday; ^∗∗^p < 0.01; ^∗∗∗^p < 0.001.*

**FIGURE 1:**
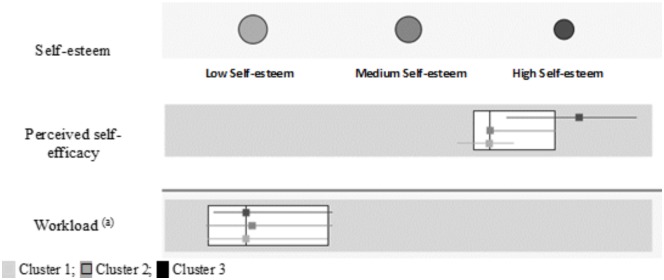
Comparison of clusters. ^(a)^Number of users attended to in a workday.

A cluster analysis was done to form the groups entering the following variables: self-esteem (low, medium, and high), perceived self-efficacy, and workload. Three groups resulted from this analysis (Figure 1), distributed as follows: 43.2% (*n* = 563) of the participants pertained to Cluster 1, 35.9% (*n* = 468) to Cluster 2, and the remaining 20.9% were in Cluster 3 (*n* = 272).

The first group (Cluster 1) was characterized by low self-esteem and means slightly below the mean of the total sample on perceived self-efficacy (*M* = 29.27) and number of users attended to (*M* = 20).

The second group (Cluster 2) included nursing professionals with a medium level of self-esteem who scored near the mean in perceived self-efficacy (*M* = 31.85), and a slightly higher mean (*M* = 21.75) in workload than the total sample.

The third group (Cluster 3) identified professionals who had high self-esteem, medium scores in perceived self-efficacy (*M* = 35.04) and were above the mean of the total sample. In this group, the mean users attended to per workday was similar to the mean for the sample (*M* = 20.93).

After classification in groups, based on the three-cluster solution, an ANOVA was performed to find out whether there were any differences in the clusters with respect to burnout. The Scheffé test was used for *post hoc* comparisons.

As observed in Table 2, there were significant differences between clusters [*F*_(2,1304)_ = 38.94; *p* < 0.001; $\eta_{p}^{2}$ = 0.05] for burnout scores. Cluster 1 is where the mean score on burnout was highest (*M* = 57.14; *SD* = 7.18), followed by Cluster 2 (*M* = 55.09; *SD* = 7.49) and, finally with the lowest mean score in burnout, Cluster 3 (*M* = 52.43; *SD* = 7.16). *Post hoc* analyses showed that the differences found among the three groups were statistically significant.

**Table 2:** Differences in burnout between groups (clusters).

	Cluster	*N*	*Mean*	*SD*	ANOVA		Difference in means
					*F*	Sig.	
Burnout	1	563	57.14	7.18	38.94	0.000	|c1-c2|^∗∗∗^|c2-c3|^∗∗∗^|c1-c3|^∗∗∗^
	2	468	55.09	7.49			
	3	272	52.43	7.16			

*Descriptive, ANOVA, and post hoc; ^∗∗∗^p < 0.001.*

### Multiple Mediation Model for Estimating Predictors and Paths of Indirect Effects of Perceived Self-Esteem and Self-Esteem on Burnout

Considering workload as the independent variable (X), and self-efficacy and self-esteem as mediating variables (M_1_: SELF-EFFICACY and M_2_: SELF-ESTEEM), the multiple mediation model was computed with burnout as the dependent variable (Y).

**FIGURE 2:**
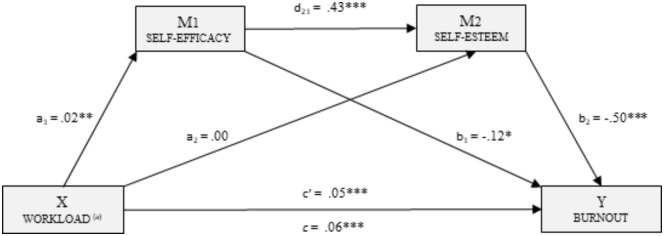
Multiple mediation model of perceived self-efficacy and self-esteem on the relationship between workload and burnout. ^(a)^Number of users attended to in a workday. ^∗^The correlation is significant at 0.05; ^∗∗^The correlation is significant at 0.01; ^∗∗∗^The correlation is significant at 0.001.

Figure 2 shows the multiple mediation model for burnout including direct, indirect and full effects.

In the first place, there was a statistically significant effect [a_1_: *B* = 0.02, *p* < 0.01] of workload (X) on perceived self-efficacy (M_1_). The second regression analysis took the second mediator (M_2_) as the result variable, and included the workload (X) and perceived self-efficacy (M_1_) variables in the equation. There was a significant effect of self-efficacy [d_21_: *B* = 0.43, *p* < 0.001] on self-esteem (M_2_), but the same was not true of self-esteem [a_2_: *B* = 0.00, *p* = 0.74].

In the third regression analysis, the effect of the independent variable and the two mediators was estimated taking burnout (Y) as the result variable. In all cases, significant effects were observed: workload [c’: *B* = 0.05, *p* < 0.001], perceived self-efficacy [b_1_: *B* = -0.12, *p* < 0.05], and self-esteem [b_2_: *B* = -0.50, *p* < 0.001]. The total effect of workload on burnout was significant [c: *B* = 0.06 *p* < 0.001].

Finally, the indirect effects were analyzed by bootstrapping, finding data supporting a significant level for Path 1 [ind_1_: X → M_1_ → Y; *B* = -0.002, *SE* = 0.001, 95% CI (-0.007, -0.000)] and Path 2 [ind_2_: X → M_1_ → M_2_ → Y; *B* = -0.004, *SE* = 0.001, 95% CI (-0.008, -0.001)]. However, the data did not support significance for Path 3 [ind_3_: X → M_2_ → Y; *B* = -0.000, *SE* = 0.002, 95% CI (-0.007, 0.004)].

## Discussion

Nursing professionals care for a large volume of patients during their workday, sometimes with an imbalance between time available to attend to their needs adequately and the workload. This workload significantly influences the health, wellbeing of the employees and the plans for working longer, generating negative feelings and job dissatisfaction because they are unable to provide quality service (Cooper et al., 2016; Van Bogaert et al., 2017). According to the Job Demands and Resources Model, workload is a powerful stressor, in addition to a predictor of burnout, through a process of worsening health (Xanthopoulou et al., 2007; Bakker et al., 2014). However, our results showed that self-efficacy and self-esteem work as buffers of the negative effects of workload on burnout. In fact, authors such as Bakker et al. (2014) and Bakker and Demerouti (2017) have shown the outstanding role of personal resources for their capacity to attenuate the negative impact of job stressors and their relationship with various positive results in the sphere of organization (e.g., engagement and job performance).

It has also been found that beliefs about self-efficacy influence self-esteem itself, increasing one’s perception of self-worth as stressful situations are overcome successfully. Bandura’s Cognitive Social Theory (1977) showed that positive beliefs in self-efficacy increase motivation to begin and maintain behavior for reaching desired goals, so successful experiences increase positive self-evaluations of self-worth and this affect the plans to work longer. Furthermore, longitudinal studies have demonstrated that there is a reciprocal relationship between self-efficacy and self-esteem over time (Caprara et al., 2010, 2013).

The mediation models show that a chain relationship may be established, in which the number of users attended to in a workday present opportunities to evaluate the degree to which professionals perceive the efficacy with which they perform their tasks, with the consequent repercussion on their self-esteem. Thus, job demands are considered challenge stressors which promote personal growth of the workers, generating positive emotions (LePine et al., 2005; Van Bogaert et al., 2017). In this light, nursing professionals consider the effort required to care for a large volume of patients to be related positively to the probability of performing the task satisfactorily, and covering this demand is also associated with positive consequences, such as the plans to work longer.

In addition to this, the results show that self-efficacy and self-esteem interact with each other, and can attenuate the negative effect of workload on burnout. Thus nursing professionals with high levels of beliefs of self-efficacy cope with the workload with effort and perseverance, contributing to maintaining optimum levels of self-esteem, and thereby, increasing their engagement with their work (Lorente et al., 2014; Ventura et al., 2015; Bajaj et al., 2016).

The evidence derived from this study transforms into relevant practical implications. For example, the important effects which personal resources such as self-efficacy or self-esteem have on job demands, wellbeing of nursing professionals and occupational health should be emphasized (Molero et al., 2018a). This is why organizations should design interventions oriented toward fostering personal resources of workers through training activities and organizational resources (e.g., redesigning job positions) to promote the satisfaction and wellbeing of their employees.

Nonetheless, the results should be considered under some limitations. The first is the method used to collect the data, which could be biased by the variance of the single method, and should therefore incorporate other qualitative methods (e.g., interviews). Second, the sample is made up of a majority of women, with the difficulty associated with generalizing the results to the entire group. Finally, with this study design, it is not possible to find out whether the data on burnout remain constant over time, and determination of the influence of time or change variables on the relationships between the variables is impeded.

As future lines of research, we suggest including other job demands (e.g., work shifts and role stress) and other psychological variables (e.g., emotional intelligence and social skills), the expectations of time to work, and variables related to work resources such as autonomy and leadership style, to complete the job resources and demands model and offer better understanding of the phenomenon. It would likewise be of interest to perform multi-level studies on burnout by the area where the nursing professionals work, so organizational preventive measures can be implemented.

## Conclusion

The main objective of this study was to evaluate the mediating role of psychological variables on the effect of workload on burnout in nursing professionals. It revealed that workload has a significant positive relationship with burnout, while self-efficacy and self-esteem act as protective variables. It was also demonstrated that workload has an indirect effect on self-esteem, mediated by beliefs about self-efficacy, and that the joint effect of self-efficacy and self-esteem can buffer the negative effect of workload on burnout. In view of all of the above, a line of research is now starting in which the analysis of the complex relationships established between the different variables and the effects of their combination, beyond the impact of isolated variables on burnout, is prioritized.

## Ethics Statement

This study was carried out in accordance with the recommendations of ‘Bioethics Committee of the University of Almería (Spain),’ with written informed consent from all subjects. All subjects gave written informed consent in accordance with the Declaration of Helsinki. The protocol was approved by the Bioethics Committee of the University of Almería (Spain).

## Author Contributions

MM and MPF contributed to bibliographic review, article writing, and data analysis. All authors contributed to researchers of the project to which the article data belong.

## Conflict of Interest Statement

The authors declare that the research was conducted in the absence of any commercial or financial relationships that could be construed as a potential conflict of interest.
